# Incisional hernia after liver transplantation: mesh-based repair and what else?

**DOI:** 10.1007/s00595-020-02162-9

**Published:** 2020-10-16

**Authors:** Aristotelis Perrakis, Dagmar Knüttel, Mirhasan Rahimli, Mihailo Andric, Roland S. Croner, Nikolaos Vassos

**Affiliations:** 1 Department of Surgery, University Hospital of Magdeburg, University of Magdeburg, Leipzigerstr. 44, 39120 Magdeburg, Germany; 2 Department of Surgery, University Hospital Erlangen, University of Erlangen-Nuremberg, Erlangen, Germany; 3 Division of Surgical Oncology, Department of Surgery, Mannheim University Medical Centre, University of Heidelberg, Mannheim, Germany

**Keywords:** Incisional hernia, Mesh, Liver transplantation

## Abstract

**Purpose:**

Incisional hernia (IH) is not uncommon after liver transplantation (LT). We investigated the long-term outcome of mesh-based hernia repair using an inlay-onlay technique.

**Methods:**

Our analysis was based on a prospective collected database of all LT recipients from our hospital over a period of 15 years. We analyzed clinical data including the period between LT and hernia development, the size and localization of the hernia, the length of in-hospital stay, immunosuppression, and postoperative morbidity, as well as follow-up data. The median follow-up period was 120 (range 12–200) months.

**Results:**

Among a total of 220 patients who underwent a collective 239 LTs, 29 (13%) were found to have an IH after a median period of 27.5 months (range 3–96 months). There were 12 (41%) men and 17 (59%) women, with a median age of 51 years. The median size of the IH was 13 cm (range 2–30 cm) and the median in-hospital stay was 6 days. Mild postoperative complications developed in seven patients, including two onlay mesh infections. One patient (3.4%) suffered recurrence.

**Conclusion:**

Mesh-based hernia repair using the inlay/onlay technique represents an effective and safe method for patients with an IH after LT, without additional risk from continuous immunosuppression.

## Introduction

Incisional hernia (IH) is a common complication after major surgery, with a reported incidence between 5 and 25% [1–5]. Risk factors for an IH after major surgery include obesity, wound infection in the immediate postoperative setting, male gender, and diabetes mellitus [6–15]. Further predisposing factors are collagen disorders, age, rapid weight loss, multiple pregnancies, chronic pulmonary disease, trauma, iatrogenic causes, and congenital disorders [12–20]. Clinical examination and ultrasonography of the abdomen are the most sensitive diagnostic tools for an IH. After its diagnosis, surgical treatment is generally indicated because of the risk of serious complications, such as incarceration and strangulation of the protruding structures. In the posttransplant setting after orthotopic liver transplantation (LT) there are specific parameters, such as ascites and the continuous administration of immunosuppression, especially corticoids, which represent additional risk factors for IH after LT [8–13, 15–29]. Τhe type of incision, performed during the LT, also plays an important role. Janssen et al. described IH after LT occurring at the site where the horizontal incision meets the vertical incision [16]. There is also a correlation caused by a disturbed balance in the collagen I to collagen III ratio. Collagen III can have less mechanical stability than collagen I [17, 18]. Moreover, the discrepancy in size between the transplant and the intraabdominal space in the right hemiabdomen, increasing mechanical strain on the wound, may be a further causal factor for the development of an IH [12, 13, 16]. IHs usually develop within the first 24 months after major surgery [14, 16, 19].

Initially, there was scepticism regarding mesh hernia repair after LT, especially using the inlay/onlay technique, because of the perceived risk of major postoperative complications in patients under immunosuppression. However, several authors report that there is no increased morbidity between mesh and non-mesh hernia repair after LT [23, 26, 29]. We report the long-term results of mesh hernia repair using the inlay/onlay technique in patients with an IH after LT, focusing on postoperative and long-term morbidity, quality of life, and recurrence rates.

## Patients and methods

### Study population

During a 15 year period, 220 patients underwent a collective 239 LTs at the Department of Surgery of University Hospital Erlangen, Germany. IH developed in 29 of these patients (13%). These 29 patients, identified from a prospective database, were the subjects of this retrospective study. The median follow-up was 120 months (range 12–200). We analyzed the demographic and clinical data, characteristics of the hernia, mode of surgical treatment, and postoperative and long-term outcomes.

### Liver transplantation

The surgical incision for LT was L-shaped and LT was performed using the piggyback technique in all patients. Patients underwent either a simultaneous portoarterial reperfusion or a portal reperfusion of the transplant. The abdomen was closed with a running suture placed through the posterior and anterior rectal sheaths. If this was not possible, absorbable mesh (Vicryl-mesh) was placed.

### Immunsuppression protocol and antibiotic prophylaxis after LT

All patients were treated initially with tacrolimus, from 36 h after transplantation, as 0.1 mg/kg twice daily, and then with methylprednisolone 500 mg in the anhepatic phase. The patients also received basiliximab (Simulect), 20 mg, in the anhepatic phase followed by a second dose of 20 mg, 4 days after transplantation. Mycophenolate mofetil (MMF), 500 mg twice daily, was given intravenously or orally from the postoperative day (POD) 5. Acute rejection was diagnosed based on histopathological examination after liver biopsy according to the Banff criteria. In 18 patients, the immunsuppressive regimen was converted to ciclosporin-based immunsuppression because of tacrolimus neurotoxicity. All recipients received broad-spectrum antimicrobial prophylaxis, consisting of antibacterial, antiviral, and antimycotic agents; with piperacilline-tazobactam for 7 days, as well as acyclovir and anidulafungin/posaconazole. Selective digestive decontamination consisted of oral amphotericin B 200 mg, three times daily until POD 21. Furthermore, high-risk patients and recipients of a cytomegalovirus (CMV)-positive donor, received preemptive antiviral treatment with ganciclovir/valganciclovir adjusted to renal function over a 6 week course. The standard laboratory work-up included hematologic and biochemical parameters. The CMV status (viral load, pp65 antigen) was examined twice a week. CMV infection was defined by the appearance of the CMV antigen polymerase chain reaction in the blood. Chest x-ray and ultrasounds of the graft were performed daily.

### Hernia repair and postoperative follow-up

All patients with an IH underwent an elective mesh-based hernia repair, under stable transplant function. After performing tensiometry, polypropylene mesh was implanted to close the hernia using the inlay/onlay technique, as described [23], and a 12Ch redon-drain was placed. None of these patients showed any sign of impaired liver function. The vast majority of patients underwent treatment with tacrolimus-based immunosuppression. In the pre- and postoperative setting, we adjusted the tacrolimus levels to within the lower range (4–6 ng/ml) to balance good graft function and a lower infection risk. Furthermore, the immunosuppressive regimen was switched to a calcineurin-inhibitor (CNI) 4 weeks before surgery for patients with (mammalian target of rapamycin) mTOR-based immunosuppression. The parameters assessed included blood lοss in the early postoperative course, postoperative morbidity and mortality, length of stay in the hospital, and relief of symptoms. During follow-up, patients underwent a clinical examination and ultrasonography of the abdomen every 3 months. Any defect in the abdominal wall was considered to be a recurrence. All patients were interviewed with the aid of an EQ5Dquestionnaire to evaluate the quality of life after surgery [30].

### Statistical analysis

Statistical analysis was performed with the statistical software SPSS for Windows (version 22.0, SPSS Inc., Chicago, IL). Continuous variables are expressed as means ± standard deviation, and differences were analyzed with the Mann–Whitney *U* test. Categorical variables were analyzed with Fisher’s exact test. A *p* value of less than 0.05 was considered significant.

## Results

### Demographic, clinical, and perioperative data

Among the 220 patients who underwent a collective 239 liver transplantations, 29 (13%) suffered an IH after a median period of 27.5 (range 3–96) months Our cohort included 12 men (41%) and 17 (59%) women, with a median age of 51 years (range 19–70 years). The main clinical symptoms were abdominal discomfort and/or recurrent pain. The diagnostic workup consisted of clinical examination and ultrasonography (US) of the abdomen. The majority of IHs developed in the first 2 years after LT. LT was indicated for acute liver failure (ALF) in five patients (17%), alcoholic cirrhosis in 11 (38%), hepatocellular carcinoma (HCC) within the Milan criteria in 6 (20.5%), primary sclerosing cholangitis (PSC) in 3 (10%), and hemochromatosis, hepatitis C cirrhosis, autoimmunhepatitis, and cholangiocellular carcinoma in 1 patient each respectively (Table 1). Τhere was no correlation between the age of the patients and the occurrence of an IH (*p* = 0.483) or between corticoid-based immunsuppressive regimen and the incidence of IH (*p* = 0.785). Patients who underwent a reoperation after LT, either for bleeding and large hematoma or for primary non-function, showed an increased risk of IH development (*p* = 0.04).

**Table 1:** Clinical and intraoperative data and postoperative outcomes

	Surgery (*n* = 29)
Age mean (years, range)	51 (19–70)
Gender (%)	
Male	12 (41)
Female	17 (59)
LT indication (%)	
Alcoholic cirrhosis	11 (38)
Hepatocellular carcinoma	6 (20.5)
Acute liver failure	5 (17)
Primary sclerosing cholangitis	3 (10.5)
Hemochromatosis	1 (3.5)
Hepatitis C cirrhosis	1 (3.5)
Autoimmune hepatitis	1 (3.5)
Cholangiocellular carcinoma	1 (3.5)
Median size of IH (cm)	13 (2–35)
Blood loss (%)	
< 200 mL	29 (100)
≥ 200 mL	0 (0)
Length of in-hospital stay/ days (range)	6 (1–15)
Complications (%)	7 (24)
Seroma	3 (10)
Subcutaneous hematoma	2 (7)
Onlay mesh infection	2 (7)
Perioperative mortality (%)	0 (0)
Relief of symptoms (%)	29 (100%)

The median size of the IH defect was 13 (range 2–35) cm. In most patients (75%), the IH developed at the site where the horizontal incision meets the vertical incision. There was no case of IH in the right lateral site of the incision. Blood loss during mesh hernia repair was less than 200 ml in all patients. The median hospital stay was 6 days (range 1–15 days) and there was no peri- or postoperative mortality in our cohort. Postoperative complications developed in seven (24%) patients, as seroma formation in three, a subcutaneous hematoma in two, and a localized infection of the onlay mesh in two. There was no case of serious deep infection or bowel fistula after the implantation of inlay/onlay mesh (Table 1). Six of these seven patients with a minor complication were managed with conservative treatment, but one patient with Crohn disease, who underwent LT for PSC, had to have the onlay mesh removed. Body mass index (BMI) > 25 kg/cm2 (*p* = 0.745), a corticoid-based immunsuppressive regimen (*p* = 0.194), age (*p* = 0.313), and size of the hernia (*p* = 0.132) were not considered predisposing factors for postoperative complications. Hernia recurrence developed in one patient (3.4%). Symptom relief was recorded for all patients (Table 2).

**Table 2:** Quality of life after incisional hernia repair (EQ 5 D)

	*n* = 29
Mobility	
No problems	29 (100%)
Moderate	0 (0%)
Immobility	0 (0%)
Self-reliance	
Full	29 (100%)
Moderate	0 (0%)
No	0 (0%)
Pain / discomfort (scale: 0–10)	
No	25 (86%)
Moderate (1–5)	4 (14%)
Extreme (6–10)	0 (0%)
Health status (mean)	
0: worst health status	95% (0–100)
100: best health status	

## Discussion

Incisional hernia, defined as the breakdown or loss of continuity of a fascial closure, is one of the most frequent complications of abdominal surgery. Patients in the posttransplant setting have a higher risk of the development of an IH, because of impairment in the wound healing processes and the higher incidence of wound infections [8, 10, 16, 22]. Furthermore, risk factors such as the presence of ascites, more than one laparotomy and acute rejection episodes play a role in the occurrence of IH after LT [12, 16, 23]. The incidence of IH in this series was 13%, which is comparable to that of other studies [10, 12, 16, 23, 24, 26, 28, 29]. Mesh-based hernia repair has been intensively discussed as the most appropriate option, considering the high recurrence rate (30–60%) after conventional suture repair [5, 16, 25–29]. Despite initial criticism because of the potential infection risk of mesh in patients under immunosuppression, the low registered complication rate led to the establishment of mesh-based hernia repair. In fact, the recurrence rate after mesh-based hernia repair is very low, whereas other considerable complications, such as wound infection and chronic pain do not represent frequent events in terms of postoperative morbidity [16, 23–26, 29]. In our collective, the fact that treatment with tacrolimus-based immunosuppression was given in a lower range without impairment of graft function, and the immunosuppression in patients with mTOR was switched to a CNI-based regimen, account for the low morbidity and recurrence rate in our patients.

The advantages of laparoscopic hernia repair as intraperitoneal onlay mesh repair (IPOM) include a shorter in-hospital stay and lower incidence of wound complications, particularly in the case of large or multiple hernia defects and in obese patients [2, 3, 21, 31]. The mesh we used for the IPOM technique is a dual-layer composite mesh (DynaMesh), composed of 88% high purity polyvinylidene fluoride (PVDF) and 12% polypropylene (PP). The size of the mesh is decided after identification of the fascial margin since the overlap has to be a minimum of 5 cm on all sides. The fixation can be achieved by transfascial sutures and clips, with tucks or glue [31]. Reported contraindications are severe adhesions and a “close-to-bone” situation, because of the high risk of severe chronic pain related to the fixation technique [31]. There is also a higher recurrence rate after repair of large IHs. Scheuerlein et al. reported a complication rate of 33% after IPOM vs. 21% after conventional mesh-based repair [31]. In our study, the mean hospital stay was 6 days, which was comparable to other studies, even to series in which patients underwent IPOM [21, 31].

Regarding postοperative morbidity, an infection around the onlay mesh developed in two patients, one of whom required its explantation. The first patient had a giant IH, 35 cm in diameter, and in the postoperative setting, a wound infection developed as a local infection around the onlay mesh, which could be treated conservatively. In this case of a larger abdominal wall defect, a component separation method could have been considered. Τhe second patient was on continuous prednisolone treatment for Crohn’s disease and the onlay mesh had to be explanted. However, there was no significant increase in postoperative morbidity among patients on a cortisone-based immunosuppressive regimen or on MMF (*p* = 0.194), as reported by others [31]. The morbidity rate and reoperation rate (for example, to remove the mesh) in our study were comparable to those in other series, which reported complication rates of 2–16% and mesh infection rates of 2.7–7.7% [11, 16, 21, 25, 26, 29, 40]. Furthermore, the hernia recurrence rate was very low (3.4%), and even lower than those in studies of patients who underwent laparoscopic hernia repair [11, 16, 25, 26, 28, 29, 31].

A limitation of the present study is its retrospective nature. Nevertheless, the data reported included all patients who underwent elective and emergency surgery with no exclusions and with a very long follow-up. Another limitation is that this study was performed in a single institution and the results obtained might not be comparable to those in other centers. However, unicentral studies have the advantage of minimizing the possible differences in surgical technique.

## Conclusion

Incisional hernia is a frequent complication after liver transplantation. Our results demonstrate that mesh hernia repair using an onlay/inlay technique is an efficient and safe method of treating patients with an IH after LT. The recurrence rate was very low and the implantation of a mesh was not associated with increased morbidity, despite the continuous use of immunosuppression. Laparoscopic mesh hernia repair is an integral part of the surgical treatment of IH, but there are still some limitations regarding its indication, especially in patients with firm adhesions and a large hernia defect, and its long-term outcomes. Therefore, proper patient selection for this treatment modality is essential. To establish the best mode of hernia repair in these patients, the results and effectiveness of each technique must be analyzed in controlled studies.
